# Yellow Fever

**Published:** 1883-08

**Authors:** 


### YELLOW FEVER.

IT IS hardly necessary to describe minutely the symptoms of a disease so well marked and so easily recognized as Yellow Fever; neither should I comply with the request of a Southern reader of the Journal to write an article upon the subject were it not that I have had considerable experience in the treatment of the disease, and may be able to make some suggestions of value to others, based on this experience.

The most important question for any community where the disease is liable to make its appearance is, whether it is *contagious* or not? I have never had the disease, though I have seen it in all its stages, and watched its course with the greatest interest, taking no precaution whatever to avoid contracting it. If the disease is highly contagious—as many physicians claim—why did I escape? For the same reason, in my opinion, that physicians usually escape disease; they don't know fear. In the hospitals of Paris, small-pox patients are distributed through the medical wards, and no precautions against the disease are taken by physicians and students who daily visit these wards. Experience and observation have convinced me that no person in sound health, and who is fearless, will take any of the so-called contagious diseases. It is only when these conditions are changed; when the brave man is worn and weary in his efforts to save others, that he becomes a prey to the disease himself. There have been many such martyrs in the fever-scourged cities of the South. An average temperature of 80 degrees, continued for two or three months, together with filthy surroundings, seem to be necessary for the development of this fearful disease. The city of Havana, in Cuba, possesses these requisites to a greater degree than any other city on this continent, and may be said, therefore, to be the home of the disease. When the scourge commences its march in the latter part of summer, it will be observed that it visits lightly or passes by such of our Southern cities as are in good sanitary condition. New Orleans was an example of the power of cleanliness to resist disease, during the few years it was under military rule, with its strict sanitary measures. Yellow fever never originates in country districts, where the essentials to its development are wanting.

It is no evidence of cowardice, but rather a duty, for a man who resides in a city that is likely to be visited by yellow fever to send his family to some healthy locality in the country. It would be rash to expose them to a scourge for which they are not responsible. No citizen long a resident in a locality whose sanitary condition is bad is in a physical condition to resist yellow fever, and to remain there after the disease has commenced its ravages would be suicidal. A far better plan, however, is to make every Southern city *fever proof*, by cleanliness and a thorough system of drainage; and then not to be alarmed at the few mild cases that may be reported. The disease would not extend far under these conditions.

It would be an assumption on the part of a *Health Journal* to discuss extendedly the symptoms and treatment of yellow fever. We leave that to medical publications. It is however, entirely within our province to suggest such measures as would not only be protective against yellow fever, but all other diseases. I will endeavor to condense these suggestions into a few simple rules that can readily be referred to when required.

*First*—See that your dwelling is clean from garret to cellar; that no piles of rubbish exist in any part of it; that the carpets are fresh and clean; that the paper on the walls is not damp and musty and in condition to absorb unhealthy emanations from sick rooms or other sources. Leave no accumulations of dirt or rags in any part of the building, and particularly in the cellar. See that the door-yard is swept and kept dry.

*Second*—Nothing is so essential to health as pure drinking water. All drinking water should be tested. The most limpid water often contains the germs of disease. Nearly fill a clean pint or quart bottle with the water to be tested, add half a teaspoonful of pure granulated sugar, cork the bottle well, and leave it in the sun for two or three days. If a slimy deposit is found in the bottle the water is unfit to drink. *Another test:* Fill a clean tumbler with the water to be tested, stir into it with a glass rod just enough *permanganate of potash* to give the water a pinkish tinge. If the tinge remains the water is pure, but if the water becomes clear in an hour or two it is impure, because the *permanganate of potash* has combined with the impurities and carried them down, leaving the water clear again.

*Third*—The greatest source of water contamination is a privy vault near the well. The safety point is from 50 to 100 feet from the well, depending on the character of the soil. But a better plan is to have a water-tight box for a vault, into which dry earth or coal ashes are thrown every day, until the box is full, when it should be carried to a distance from any habitation and emptied.

*Fourth*—Avoid remaining long with damp feet or wet clothing.

*Fifth*—Any person to be healthy should have at least one movement of the bowels every twenty-four hours. This may be secured by proper food and exercise.

*Sixth*—Some persons can get along with seven hours sleep a day; eight hours are better, and for children nine or ten hours.

*Seventh*—Avoid great fatigue. Nothing more surely saps the foundation of health and shortens life as overwork of mind or body.

*Eighth*—Don't fret. Fretting is the first stage of insanity, and displays of anger and scolding are neither attractive nor conducive to health. Fretting, scolding and *fits* of anger, age the victim in appearance, and actually shorten the natural term of life.

---

The white perch of the Ohio are noted for the musical sounds they make. The sound is much like that produced by a silk thread placed in a window where the wind blows across it.

A French surgeon says that on chloroforming some mice and lifting them by their tails, they tried to bite, but on laying them again in a horizontal position they resumed insensibility. Acting on this hint, when a patient showed signs of collapse under a dose of chloroform, he dropped the patient's head over the bedside and raised the feet quite high. The patient at once became conscious; when laid straight on the bed he became insensible again, and a return to lowering the head and raising the feet for ten minutes was required to counteract the chloroform. It is thought that by this treatment anæsthetics may be used with great safety.

